# Supplementary material for: Identification of a Novel HBV Encoded miRNA Using Next Generation Sequencing
Source: Viruses. 2022 Jun 5;14(6):1223. doi: 10.3390/v14061223 (PMC9228518; doi:10.3390/v14061223)
Supplement: Supplementary file 1 [file viruses-14-01223-s001.zip › Table S1 List of Primers.pdf]

Supplementary Table S1. List of Primers

miRNA	Sequence
Oligo-dT adapter primer	5'-CATAGACCTGAATGGCGGTAAGGGTGTGGTAGGCGAGACA<br>TTTTTTTTTTTTTTTTTTTT-3'
Universal reverse primer	5'-GCATAGACCTGAATGGCGGTA-3'
IFNa_Forward	5'-GCTTTACTGATGGTCCTGGTGGTG-3'
IFNa_Reverse	5'-GAGATTCTGCTCATTTGTGCCAG-3'
IFNb_Forward	5'-GAATGGGAGGCTTGAATACTGCCT-3'
IFNb_Reverse	5'-TAGCAAAGATGTTCTGGAGCATCTC-3'
RSAD2_Forward	5'-AAGTCCATCCTGGATGTTGGTG-3'
RSAD2_Reverse	5'-TTCCGCTCTACCAATCCAGCT-3'
ISG15_Forward	5'-CATCTTTGCCAGTACAGGAGCT-3'
ISG15_Reverse	5'-ACACCTGGAATTCGTTGCC-3'
IFI44L_Forward	5'-ATGTTCAGCTGTACCCTCCCAC-3'
IFI44L_Reverse	5'-TGCCTACACTGCACTTCCTGTC-3'
IFI27_Forward	5'-TAGCAGCCAAGATGATGTCCG-3'
IFI27_Reverse	5'-AGAGTCCAGTTGCTCCCAGTGA-3'
HBV-miR-A_Stemloop	5'-GTCGTATCCAGTGCAGGGTCCGAGGTATTCGCACTGGATACGACAGAGAA-3'

HBV-miR-A _ Forward	5'-GTATACGACTCGTGGTGGACT-3'
HBV-miR-B_Stemloop	5'-GTCGTATCCAGTGCAGGGTCCGAGGTATTCGCACTGGATACGACCCAAAG-3'
HBV-miR-B _ Forward	5'-GTATACACCAATTTTCTTTTG-3'
HBV-miR-C_Stemloop	5'-GTCGTATCCAGTGCAGGGTCCGAGGTATTCGCACTGGATACGACAAGGTT-3'
HBV-miR-C_ Forward	5'-GTATACCAGCGCATGCGTGGA-3'
Universal_Reverse	5'-GTGCAGGGTCCGAGGT-3'
IFNA4_Forward	5'-TGCACAGAGCAAAGTCTTCA-3'
IFNA4_Reverse	5'-AAAAGGACAGGGCCATTGGG-3'
IFNA17_Forward	5'-GATCTGCCTCAGACCCACAG-3'
IFNA17_Reverse	5'-TCCTCCTGGGGAAGTCCAAA-3'
RAD50_Forward	5'-TTTGGTTGGACCCAATGGGG-3'
RAD50_Reverse	5'-CAGGAGGGAAATCTCCAGTACAA-3'
JMJD6_Forward	5'-TTGGACCCGGCACAACTACTA-3'
JMJD6_Reverse	5'-TCTGCCCTTTCCACGTTATCC-3'
GOLGA6A_Forward	5'-GAGCCCTGGTATTCCAGCAG-3'
GOLGA6A_Reverse	5'-TTGTCACTGAGCTTGACTCCA-3'
METTL21A_Forward	5'-GAGGAGACCACGGAATTTGGG-3'
METTL21A_Reverse	5'-CAGGTATGTGGAAAGAACGATGG-3'
SCAF11_Forward	5'-ACTGTATGTACCCTAAATATGGGAG-3'
SCAF11_Reverse	5'-AGCCTCACTGTACAACAGACC-3'

SRSF1_Forward	5'-TCGCGACGGCTATGATTACG-3'
SRSF1_Reverse	5'-ACTTGGAGGCAGTCCAGAGA-3'
EXD2_Forward	5'-GATTATGGCCTCGTTGTTAGGG-3'
EXD2_Reverse	5'-ACTTCAGGCTAAGCCCATTACA-3'
TMEM120B_Forward	5'-CTTACACTCCAGAGGTGCAAAC-3'
TMEM120B_Reverse	5'-CGCTCCTTGATGTTCGCTG-3'
LARP1B_Forward	5'-GCCAACACCAAGTGAATTAGTGA-3'
LARP1B_Reverse	5'-TTTGTTTCCCGGTTTTCTTTGC-3'
ZNF275_Forward	5'-TCTTTTGGGCGTTCCTGTTTT-3'
ZNF275_Reverse	5'-GTCTGACACCAGTAGCACTTG-3'
FSCB_Forward	5'-ATGGTAGGCAAATCCCAGCAA-3'
FSCB_Reverse	5'-GGGTAGCTTTGGGGCTAGATG-3'
WIPF2_Forward	5'-TGTCCGGTCTTTCTTGGATGA-3'
WIPF2_Reverse	5'-GGCAGCTCGGTTTGTTTTGC-3'
FCGRT_Forward	5'-GATGAGCACCACTACTGCTG-3'
FCGRT_Reverse	5'-CAAGACACCGATGACGATTCC-3'
EIF5A2_Forward	5'-GGACGACCATGCAAAATAGTGG-3'
EIF5A2_Reverse	5'-TGCCCGTGAAAATATCAATTCCA-3'
SHQ1_Forward	5'-CAAGTTCTACGCCAAGCCATA-3'
SHQ1_Reverse	5'-TGGGCAGGCGAATGGTAAAAA-3'

PET117_Forward	5'-CTAGGAGCTCGAAGGTGGTG-3'
PET117_Reverse	5'-GATAACTCCGTCACGAAGCCT-3'
