# Supplementary material for: Identification of a Novel HBV Encoded miRNA Using Next Generation Sequencing
Source: Viruses. 2022 Jun 5;14(6):1223. doi: 10.3390/v14061223 (PMC9228518; doi:10.3390/v14061223)
Supplement: Supplementary file 1 [file viruses-14-01223-s001.zip › Table S2 Potentially gene targeted of HBV-miR-6.pdf]

Supplementary Table S2. Potentially gene targeted of HBV-miR-6.

Target Rank	Target Score	Gene Symbol	Gene Description
1	92	GOLGA6A	Golgin A6 Family Member A
2	88	METTL21A	Methyltransferase 21A
3	85	SLC12A1	Solute Carrier Family 12 Member 1
4	83	GOLGA6C	Golgin A6 Family Member C
5	82	SCAF11	SR-Related CTD Associated Factor 11
6	67	SRSF1	Serine And Arginine Rich Splicing Factor 1
7	66	EXD2	Exonuclease 3'-5' Domain Containing 2
8	65	THEM120B	Transmembrane Protein 120B
9	64	LARP1B	La Ribonucleoprotein 1B
10	62	ZNF275	Zinc Finger Protein 275
11	61	RAD50	RAD50 Double Strand Break Repair Protein
12	61	FSCB	Fibrous Sheath CABYR Binding Protein
13	58	WIPF2	WAS/WASL Interacting Protein Family Member 2
14	58	FCGRT	Fc Gamma Receptor And Transporte
15	57	EIF5A2	Eukaryotic Translation Initiation Factor 5A2
16	55	MAN2B2	Mannosidase Alpha Class 2B Member 2
17	55	SLC36A2	Solute Carrier Family 36 Member 2
18	55	JMJD6	Jumonji Domain Containing 6
19	54	SHQ1	SHQ1, H/ACA Ribonucleoprotein Assembly Factor
20	53	PET117	PET117 Cytochrome C Oxidase Chaperon
21	51	IFNA4	Interferon Alpha 4
22	51	IFNA17	Interferon Alpha 17
23	50	NUBPL	nucleotide-binding protein-like
24	50	CRAMP1	Cramped Chromatin Regulator Homolog 1
25	50	ATPSCKMT	ATP Synthase C Subunit Lysine N-Methyltransferase
